# Supplementary material for: Energy Substrate Transporters in High-Grade Ovarian Cancer: Gene Expression and Clinical Implications
Source: Int J Mol Sci. 2022 Aug 11;23(16):8968. doi: 10.3390/ijms23168968 (PMC9408757; doi:10.3390/ijms23168968)
Supplement: Supplementary file 1 [file ijms-23-08968-s001.zip › ijms-1820635-supplementary.pdf]

Supplementary Material

Energy Substrate Transporters In High-Grade Ovarian Cancer - Gene Expression And Clinical Implications

Marta Baczewska, Elżbieta Supruniuk, Klaudia Bojczuk, Paweł Guzik, Patrycja Milewska, Katarzyna Konończuk, Jakub Dobroch, Adrian Chabowski and Paweł Knapp

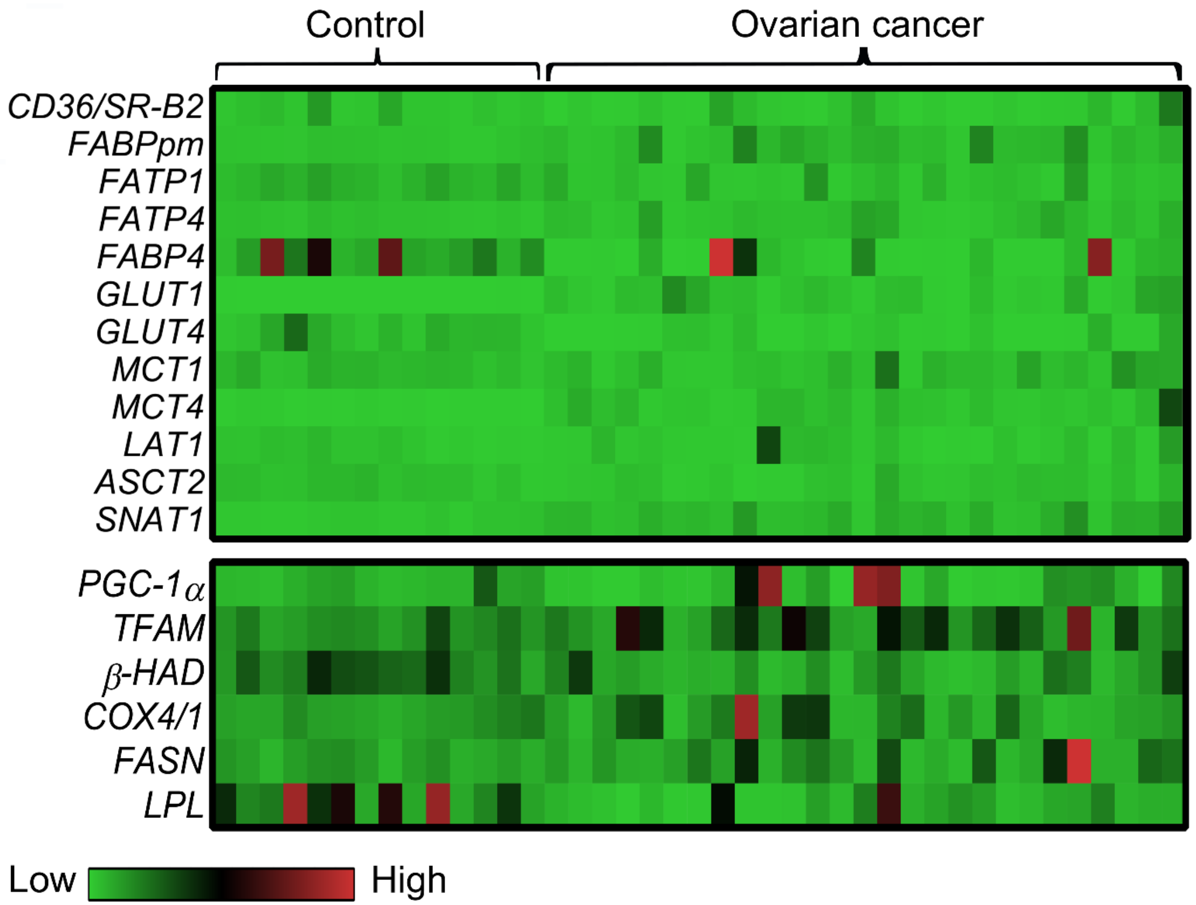

Figure S1. Heat map of relative gene expressions in control and ovarian cancer tissue.

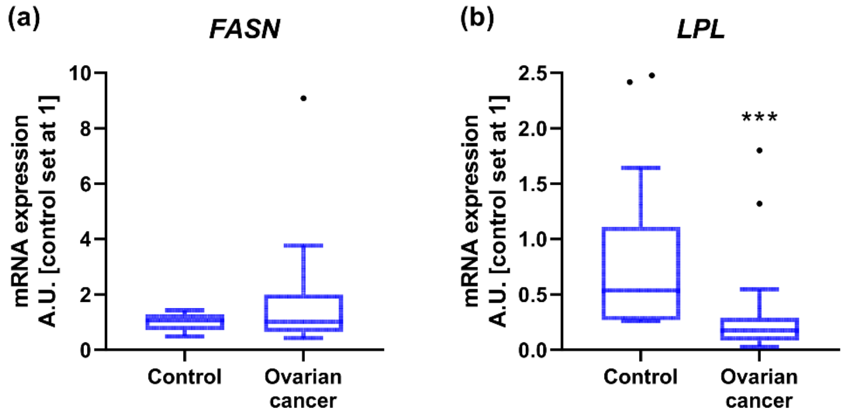

Figure S2. Transcript levels of (**a**) fatty acid synthase and (**b**) lipoprotein lipase in ovarian control (n=14) and cancer tissue (n=27). The measurements were made in duplicate, and the arithmetic means were used for subsequent investigation. The results are expressed in arbitrary units with control set as 1 and presented as median (min to max) value. *** $p < 0.001$.

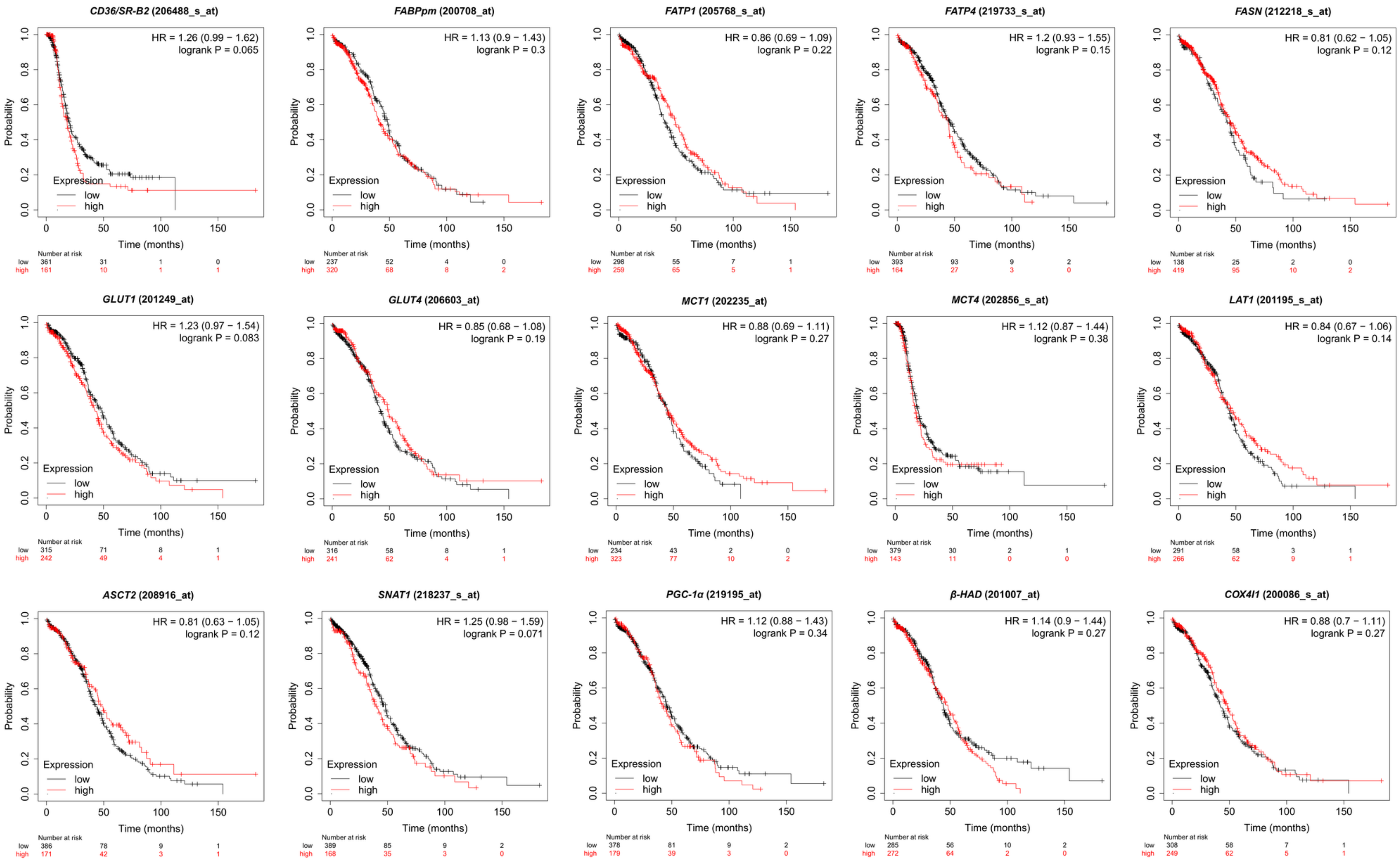

Figure S3. Kaplan-Meier curve analysis of overall survival comparing high and low levels of the metabolism-associated genes.

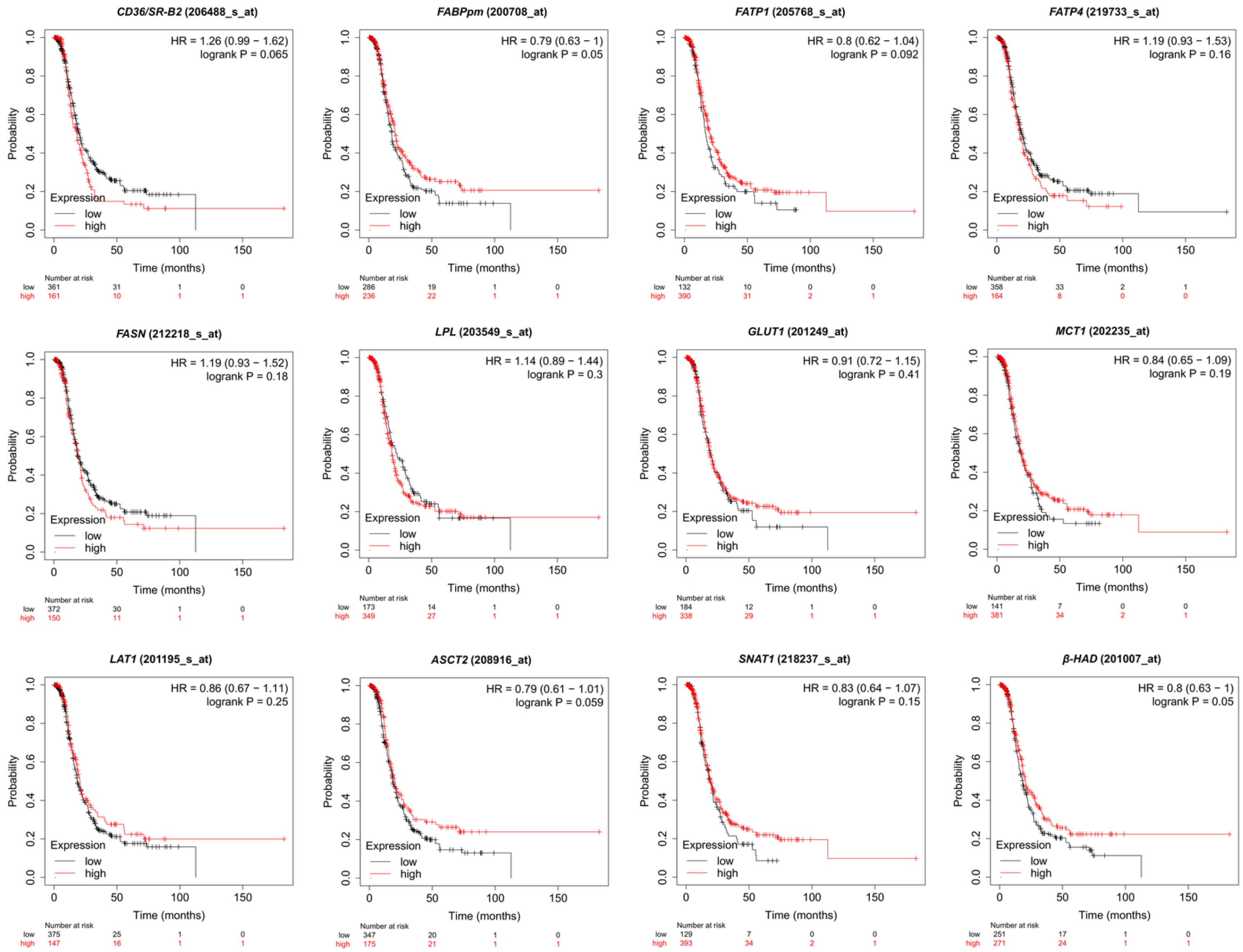

Figure S4. Kaplan-Meier curve analysis of progression-free survival comparing high and low levels of the metabolism-associated genes.

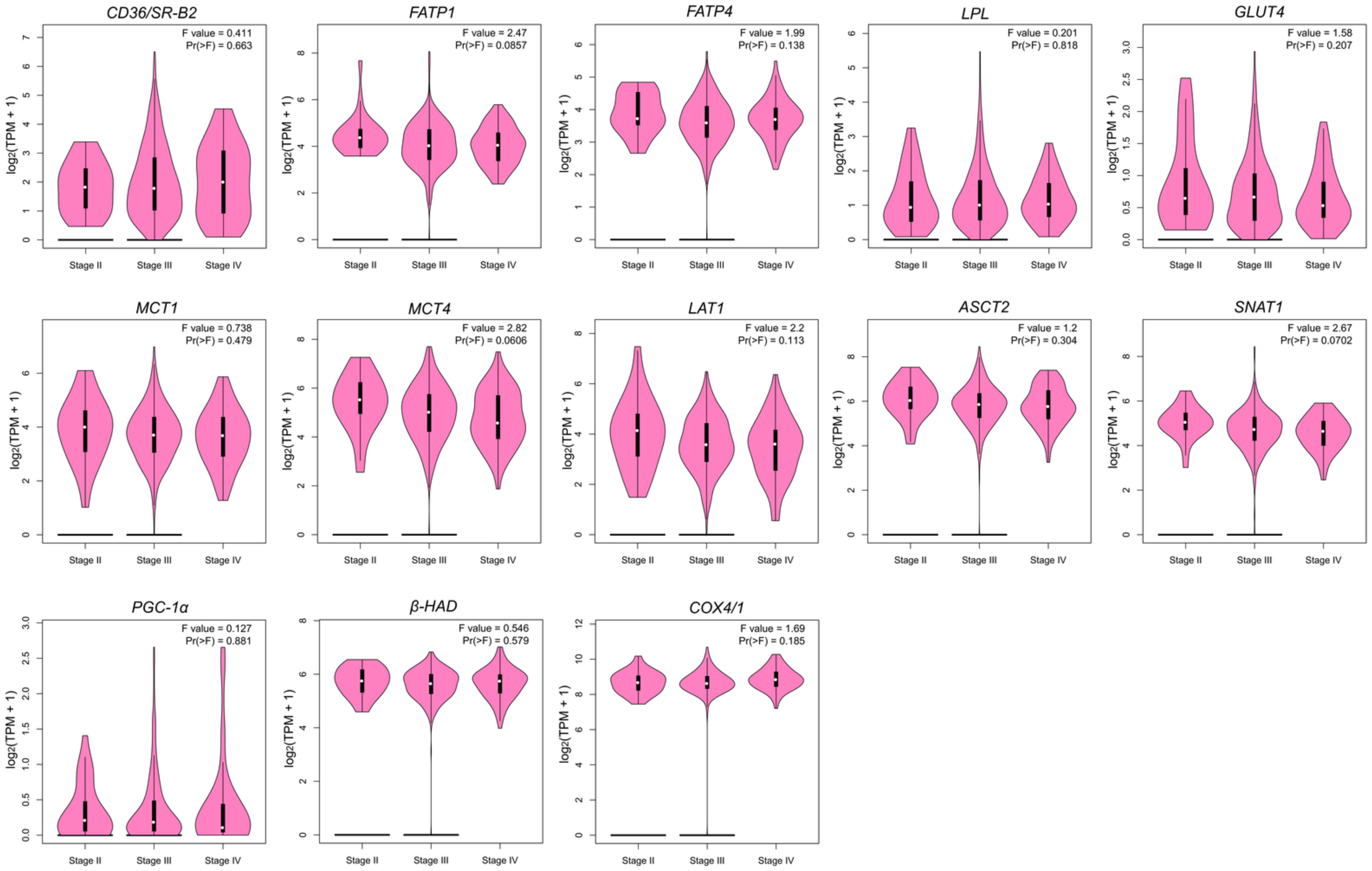

Figure S5. The violin plots depicting the correlations of metabolism-related genes with clinical stage.

Table S1. Correlations between the expression of energy substrate transporters and mitochondrial genes with BMI, plasma glucose concentration and tumor volume in ovarian cancer tissue. The relationships between the analysed parameters were assayed with Spearman correlation coefficient.

**Gene**	**BMI**		**Plasma glucose level**		**Tumor volume**	
	**r**	**p**	**r**	**p**	**r**	**p**
CD36/SR-B2	-0.0519	0.797	-0.3789	0.121	0.1374	0.494
FABPpm	0.4542	**0.017**	-0.3758	0.124	0.033	0.87
FATP1	0.0684	0.735	0.1915	0.446	0.3267	0.096
FATP4	0.2381	0.232	-0.2174	0.386	0.178	0.374
FABP4	0.235	0.238	-0.4451	0.064	-0.1197	0.552
GLUT1	-0.0226	0.911	0.5559	**0.017**	0.1206	0.549
GLUT4	0.3333	0.089	0.3509	0.153	0.1777	0.375
MCT1	0.0122	0.952	-0.1542	0.541	0.2553	0.199
MCT4	-0.2349	0.248	-0.1201	0.635	0.3084	0.125
LAT1	-0.0232	0.909	-0.4089	0.092	0.4238	**0.028**
ASCT2	0.0232	0.909	0.2039	0.417	0.2602	0.19
SNAT1	0.2418	0.224	-0.028	0.912	0.1423	0.479
PGC-1α	0.3767	0.053	-0.1864	0.459	0.2769	0.162
TFAM	0.0714	0.723	0.1128	0.656	0.0394	0.845
β-HAD	-0.0665	0.742	0.1491	0.555	0.1292	0.521
COX4/1	0.1558	0.438	-0.1097	0.665	-0.0293	0.885
FASN	0.1893	0.344	0.0714	0.778	-0.1047	0.603
LPL	0.1838	0.359	-0.1046	0.679	-0.1035	0.607
